# Secondary synovial chondromatosis of the subacromial subdeltoid bursa with coexisting glenohumeral osteoarthritis: Case report

**DOI:** 10.1097/MD.0000000000027796

**Published:** 2021-11-24

**Authors:** Hyun June Lee, Weoncheol Han, Kyungil Kim

**Affiliations:** a Department of Orthopedic Surgery, Wonkwang University Sanbon Hospital, Gunpo, Republic of Korea; b Department of Pathology, Wonkwang University Sanbon Hospital, Gunpo, Republic of Korea.

**Keywords:** arthroplasty, bursa, case report, osteoarthritis, shoulder, synovial chondromatosis

## Abstract

**Rationale:**

Synovial chondromatosis of the shoulder joint is uncommon; this condition usually affects the knee joint and the hip joint. Lesions of multiple chondral nodules form in the synovium and are usually found within the joint capsule. Treatment of synovial chondromatosis consists of loose body removal and synovectomy. In synovial chondromatosis of the shoulder, arthroscopic loose body removal and synovectomy have been reported with good outcomes. Arthroplasty can be a treatment option when osteoarthritis co-occurs at the affected joint. Since incidence of glenohumeral joint osteoarthritis is low compared to osteoarthritis of the knee or hip joints, reports of shoulder synovial chondromatosis treated with arthroplasty are scarce.

**Patient concerns:**

A 79-year-old woman presented with right shoulder pain with loss of motion for several years without a history of trauma.

**Diagnoses:**

Degenerative changes in the humeral head and glenoid were noted and multiple loose bodies were found in the subdeltoid bursa, and the subacromial bursa. The pathology of loose bodies showed degenerated cartilage tissue and some bony components. Characteristic concentric rings of calcification were observed, indicative of secondary synovial chondromatosis. The diagnosis was secondary synovial chondromatosis of the subacromial subdeltoid bursa with coexisting glenohumeral osteoarthritis.

**Interventions:**

The patient was treated with loose body removal, extensive synovectomy, bursectomy and reverse total shoulder arthroplasty.

**Outcomes:**

Visual analog scale for shoulder pain, range of motion of shoulder joint had improved demonstrating a good short-term outcome and there was no radiographic evidence of disease recurrence.

**Lessons:**

In synovial chondromatosis of the shoulder, loose bodies may form in the bursa. In combination with degenerative osteoarthritis of the glenohumeral joint, arthroplasty is a viable option.

## 1 Introduction

Synovial chondromatosis is a proliferative disease of the synovium characterized by multiple cartilaginous loose bodies formed in the joint as a result of synovial connective tissue chondrometaplasia.^[1]^ It is usually a single joint disease that is most likely to affect a large joint, such as the knee or hip joint, in males aged 30 to 50 years.^[2,3]^

The etiology of synovial chondromatosis is yet unknown but is classified as a result of primary or secondary causes. In primary synovial chondromatosis, chondrometaplasia of the synovium forms intra-articular loose bodies without any other cause. Conversely, in secondary synovial chondromatosis, loose bodies originate from osteophytes or osteochondral lesions.^[2]^

Treatment consists of loose body removal and synovectomy, and the recurrence rate is generally reported to be low. Arthroplasty can be an option when degenerative arthritis of the joint is co-occurring. There has been only 2 previous case report of shoulder arthroplasty as a treatment for synovial chondromatosis combined with degenerative arthropathy of the glenohumeral joint.^[4,5]^

We report a case and the clinical results of secondary synovial chondromatosis of the subacromial subdeltoid bursa with coexisting glenohumeral joint osteoarthritis treated with loose body removal, synovectomy and reverse total shoulder arthroplasty.

## 2 Case presentation

A 79-year-old woman presented with right shoulder pain with loss of motion for several years without a history of trauma; the pain had worsened 3 months prior. Pain developed not only with motion but also at rest and was especially severe at night, causing sleep disturbance. The patient-reported visual analog scale for shoulder pain was described as 9 out of a possible 10. The range of motion limitation was noted to be 90 degrees forward flexion, 10 degrees of external rotation and internal rotation to the level of L5.

**Figure 1:**
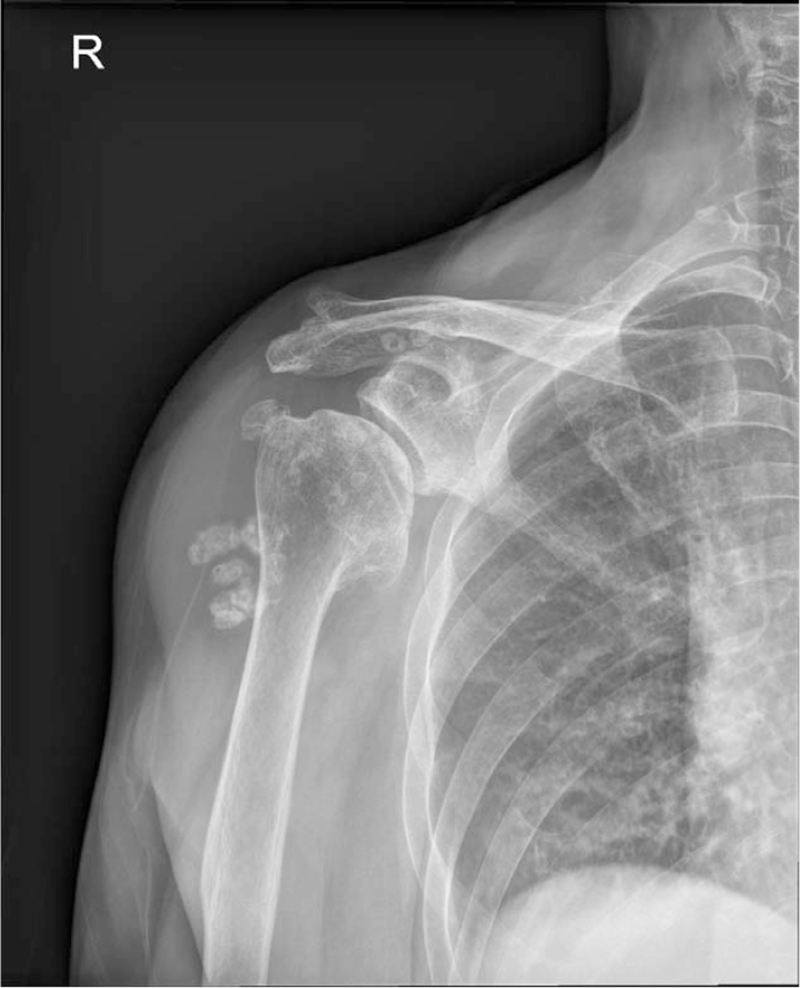
Initial shoulder radiograph shows joint space narrowing and subchondral sclerosis of the glenohumeral joint. Loose bodies were located in the subdeltoid bursa and subacromial bursal area.

**Figure 2:**
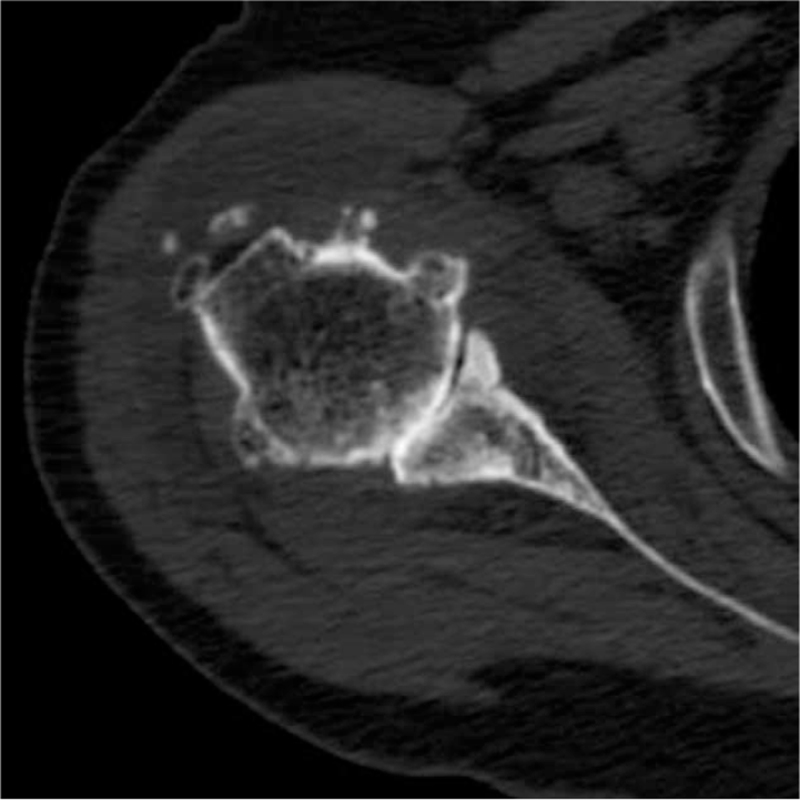
Computerized tomography scan demonstrating humeral head flattening with large osteophytes and glenoid wear from center-to-posterior.

**Figure 3:**
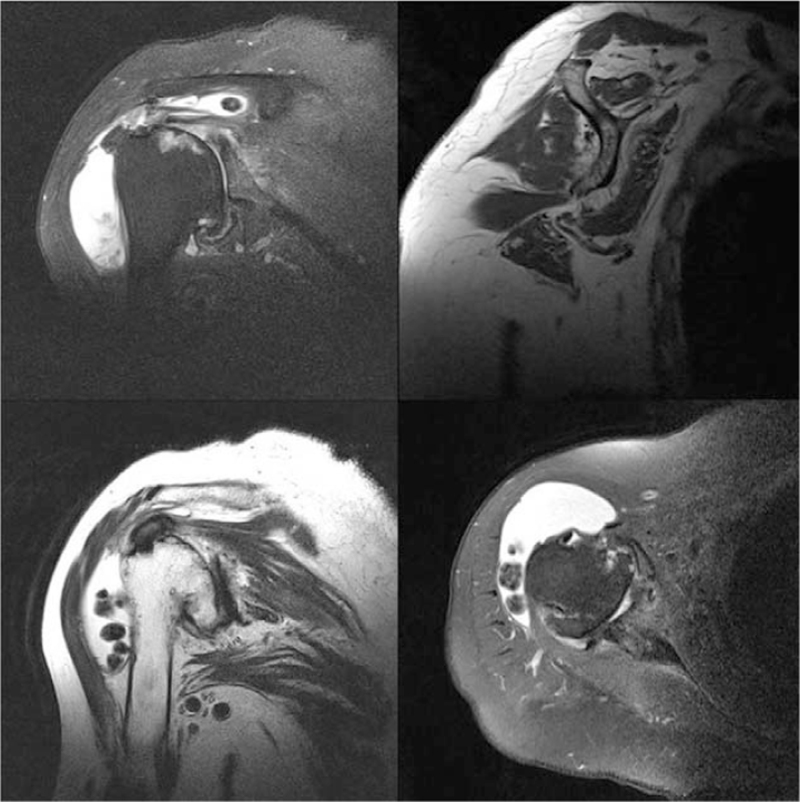
Magnetic resonance imaging demonstrating a full-thickness tear and retraction of the supraspinatus and partial infraspinatus tendon with diffuse atrophic changes in the muscle. Large amounts of fluid and multiple similarly sized loose bodies are seen in the subacromial subdeltoid bursa. Hypertrophy of the synovium is also seen.

Degenerative changes in the glenohumeral joint and multiple loose bodies were found in the subdeltoid bursa, and the subacromial bursa area was seen on plain radiographs (Fig. 1). Flattening of the humeral head with osteophytes and center-to-posterior glenoid wear were found on computed tomography (Fig. 2). Magnetic resonance imaging demonstrated rupture and retraction of the supraspinatus and infraspinatus with atrophy, a large amount of fluid with multiple similar-sized loose bodies in the bursa and thickening of the synovial membrane (Fig. 3).

**Figure 4:**
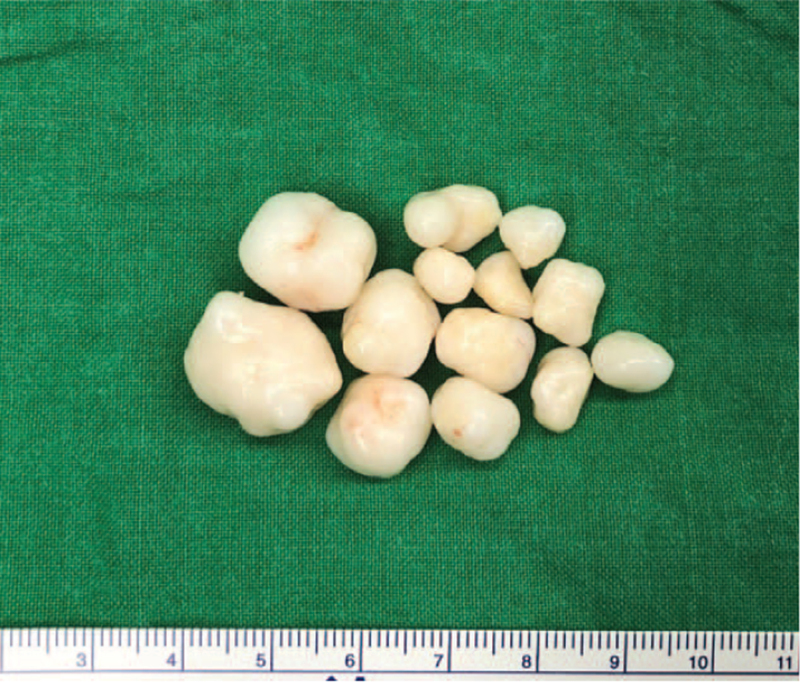
Multiple oval-shaped, hard-textured, pale white translucent glassy loose bodies found inside the bursa.

**Figure 5:**
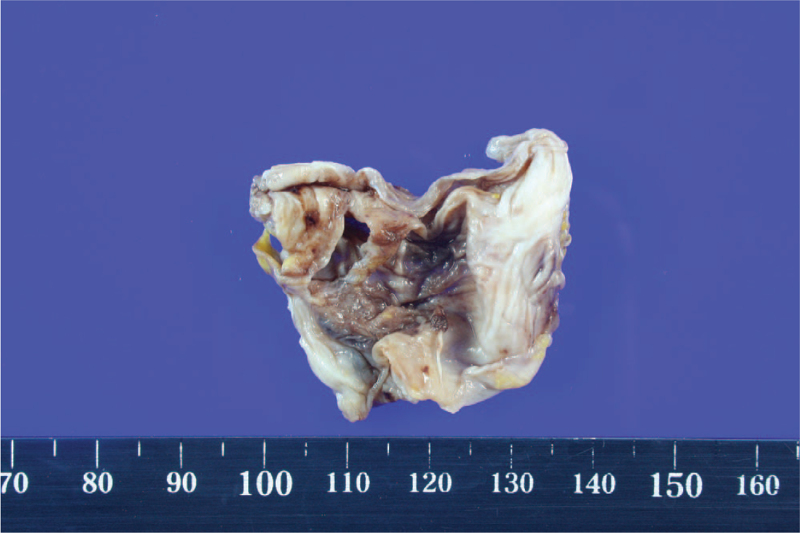
Gross photo of excised bursal synovium. The hypertrophic synovium is thick on gross exam.

**Figure 6:**
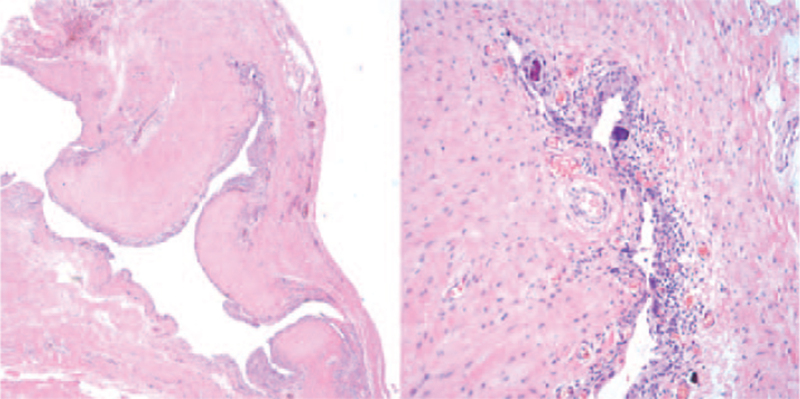
The pathology of the bursal synovium shows chronic inflammation with synovial hypertrophy, monocyte infiltration, endothelial proliferation, and dystrophic calcification (hematoxylin and eosin stain, original magnification ×40 and ×100).

**Figure 7:**
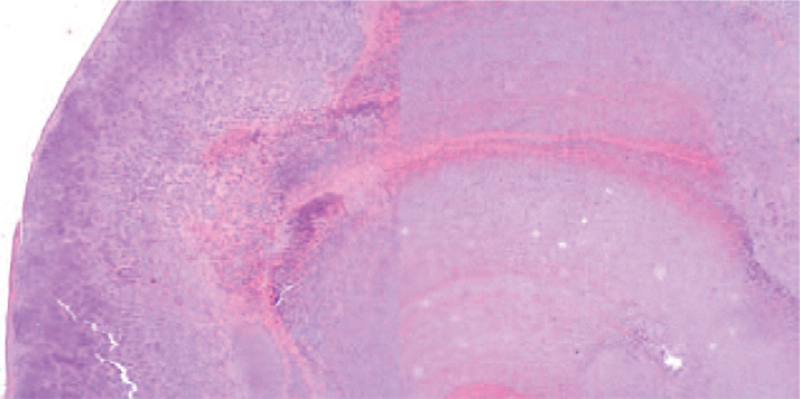
The pathology of loose bodies shows degenerated cartilage tissue and some bony components. Characteristic concentric rings of calcification found in secondary synovial chondromatosis are also seen (hematoxylin and eosin stain, original magnification ×40).

Under general anesthesia, reverse total shoulder arthroplasty was performed after loose body removal and extensive synovectomy by a deltopectoral approach in a beach chair position. The bursa under the deltoid muscle was largely inflated, and with incision, a large amount of clear, yellowish fluid gushed out. Multiple oval-shaped, hard, pale white translucent glassy-appearing loose bodies were found in the bursa (Fig. 4). Extensive synovectomy was performed since hyperplasia and inflammatory changes in the synovium were observed while removing the loose bodies (Fig. 5). Degenerative changes in the humeral head and glenoid were noted, accompanied by a full-thickness tear with retraction of the supraspinatus and infraspinatus tendons. Reverse total shoulder arthroplasty (Lima Corporate, Systema Multiplana Randelli reverse) was performed considering the patient's age and condition of the rotator cuff. Bursal fluid was sent for bacterial culture and Gram staining. Bursal synovium and loose bodies were sent for pathology. No bacteria growth was found. The pathology of the bursal synovium showed chronic inflammation with synovial hypertrophy, monocyte infiltration, endothelial proliferation, and dystrophic calcification (Fig. 6). The pathology of loose bodies showed degenerated cartilage tissue and some bony components. Characteristic concentric rings of calcification were observed, indicative of secondary synovial chondromatosis (Fig. 7).

**Figure 8:**
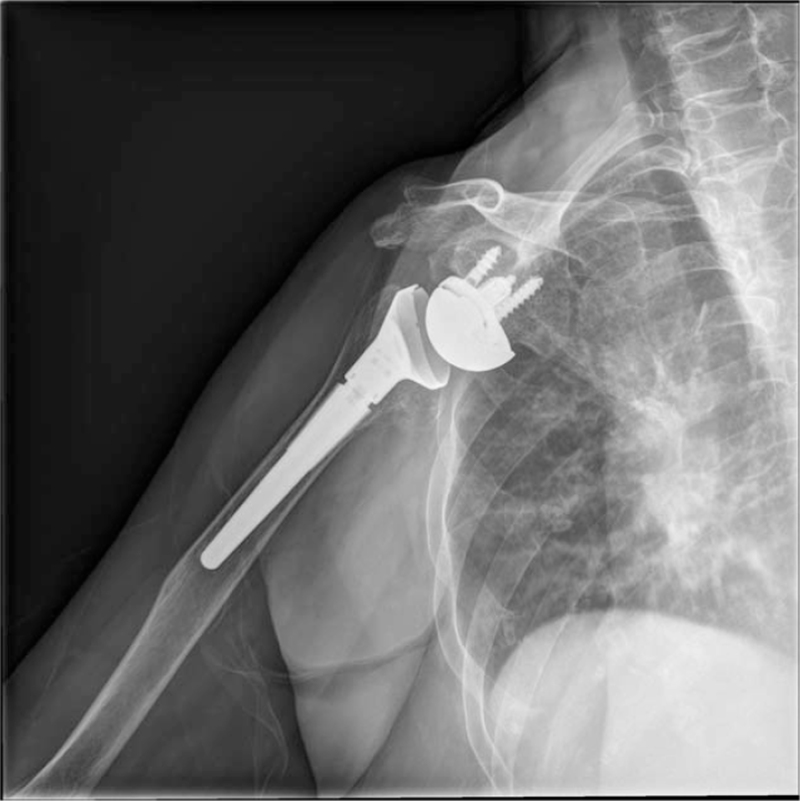
Follow-up shoulder radiograph taken 15 mo after surgery show no radiographic evidence of disease recurrence.

Six months after surgery, the patient's range of motion had improved by 140 degrees forward flexion, 30 degrees of external rotation and internal rotation to the level of the thoracolumbar junction. The patient-reported visual analog scale for shoulder pain was described as 0 to 1 out of 10 with no limitation of daily activities. Fifteen months after surgery, follow-up plain radiograph (Fig. 8) demonstrated no radiolucency around implant and no subsidence of humeral stem. New loose body formation was not seen. There was no evidence of recurrence of synovial chondromatosis.

## 3 Discussion

The etiology of synovial chondromatosis is not well understood. Trauma and infection are suggested causes, with resulting absorption of pieces of separated articular cartilage in the synovium, causing chondrometaplasia.^[6]^ Synovial chondromatosis was first described by Jaffe^[1]^ in 1958 as loose bodies in the synovium consisting of metaplastic chondroid tissue. Milgram^[2]^ classified the loose bodies by origin, as primary from synovial proliferation or secondary from osteophytes or osteochondral fractures. In addition, Milgram^[2]^ described 3 stages of synovial chondromatosis, early, transitional and late stages: active synovium without loose bodies in the early stage, active synovium with loose bodies in the transitional stage, and only loose bodies without active synovium in the late stage. Villacin et al^[7]^ reported histologic criteria for primary and secondary synovial chondromatosis, which have been shown to correlate with clinical results. Compared to primary chondromatosis, in secondary synovial chondromatosis, loose bodies and synovium may include fragments of articular cartilage, subchondral bone and fibrin. Cells are evenly distributed in the metaplastic cartilage. The pattern of calcification forms a zonal “ring-like” calcification. Enchondral ossification occurs early in regular shape.^[7,8]^

The histology of our patient showed loose bodies consisting of degenerative cartilaginous tissue and some bony components, evenly distributed cells in the metaplastic cartilage, and zonal “ring-like” calcification corresponding to secondary synovial chondromatosis. Active synovium with monocyte infiltration, endothelial proliferation and dystrophic calcification with loose bodies corresponded to the transitional stage of synovial chondromatosis.

Generally, a patient with similar characteristics may be more likely to diagnosed with secondary synovial chondromatosis caused by degenerative osteoarthritis of the shoulder joint. Secondary synovial chondromatosis originating from separated osteophytes or osteochondral fracture fragments caused by degenerative osteoarthritis progression of the glenohumeral joint usually forms within the joint capsule. There have been no reports of secondary synovial chondromatosis of the subacromial subdeltoid bursa with degenerative osteoarthritis of the glenohumeral joint treated with arthroplasty. Unlike the case reported by Kreines et al,^[4]^ in our patient, synovial chondromatosis occurred in the subacromial subdeltoid bursa, not within the joint capsule, with the histologic findings of secondary synovial chondromatosis. Degenerative osteoarthritis and secondary synovial chondromatosis occurred in the same shoulder joint, but there was a difference in the actual space, intra-articular space and bursal space. Therefore, degenerative osteoarthritis of the glenohumeral joint might not be the direct origin of secondary synovial chondromatosis of the subacromial subdeltoid bursa in our patient. Since the condition does not directly originate from separated osteophytes of osteochondral fracture fragments, other factors may have affected the bursal synovium and cause synovial chondromatosis. Conversely, synovial chondromatosis of the subacromial subdeltoid bursa affected the development of degenerative osteoarthritis of the glenohumeral joint.

Kamineni et al^[9]^ described how early removal of intra-articular loose bodies can prevent the progression of arthritis. There is no doubt that loose body removal is an effective treatment for synovial chondromatosis, but whether additional extensive synovectomy is necessary to prevent recurrence is still controversial. Jeffreys^[10]^ stated that loose body removal alone can achieve good outcomes. Milgram^[2]^ described synovial chondromatosis as a self-limiting disease; therefore, in the late stage with only loose bodies and without active lesions in the synovium, loose body removal alone without synovectomy would be satisfactory. Shpitzer et al^[11]^ reported that there was no difference between loose body removal only and additional synovectomy. Kamineni et al^[9]^ noted that additional synovectomy is not always necessary for treatment of synovial chondromatosis of the elbow joint. Conversely, Ogilvie-Harris and Saleh^[12]^ recommended that synovectomy (arthroscopic synovectomy) should be combined with loose body removal. Richman and Rose^[13]^ also concluded that arthroscopic synovectomy should be combined with loose body removal. There are no long-term follow-up results of shoulder synovial chondromatosis treated with arthroplasty, so comparison is difficult, but synovitis recurrence was reported in 2 out of 11 cases of knee synovial chondromatosis treated with arthroplasty, requiring additional synovectomy.^[14]^ The recurrence rate of primary synovial chondromatosis is reported to be up to 60%, resembling the patterns of a benign tumor. Conversely, secondary synovial chondromatosis tends to be less aggressive.^[7]^ Therefore, in primary synovial chondromatosis, additional synovectomy combined with loose body removal is necessary. In the early and transitional stages of synovial chondromatosis, since the synovium is active, additional synovectomy should be combined with loose body removal. However, distinguishing primary vs secondary synovial chondromatosis prior to histologic evaluation of loose bodies is difficult. Identifying the stage prior to histologic evaluation of the synovium is also difficult. Therefore, the authors suggest that regardless of the origin and stage, synovectomy to possible extent be considered necessary. For example, if loose body removal is performed under arthroscopic procedures, arthroscopic synovectomy should be performed to the possible extent.

## 4 Conclusion

In synovial chondromatosis of the shoulder, loose bodies may form in the bursa. In combination with degenerative osteoarthritis of the glenohumeral joint, arthroplasty is a viable option. Our patient showed an improved visual analog scale for shoulder pain and range of motion with short-term follow-up. Pain, range of motion, and component longevity could change due to synovial chondromatosis recurrence. Long-term follow-up of our patient and comparison with other cases will be needed in future studies.

## Author contributions

**Conceptualization:** Kyungil Kim.

**Data curation:** Hyun June Lee, Weoncheol Han, Kyungil Kim.

**Formal analysis:** Kyungil Kim.

**Investigation:** Kyungil Kim.

**Resources:** Kyungil Kim.

**Supervision:** Kyungil Kim.

**Visualization:** Kyungil Kim.

**Writing – original draft:** Hyun June Lee, Kyungil Kim.

**Writing – review & editing:** Kyungil Kim.
